# Zebrafish as a Novel Vertebrate Model To Dissect Enterococcal Pathogenesis

**DOI:** 10.1128/IAI.00976-13

**Published:** 2013-11

**Authors:** Tomasz K. Prajsnar, Stephen A. Renshaw, Nikolay V. Ogryzko, Simon J. Foster, Pascale Serror, Stéphane Mesnage

**Affiliations:** Krebs Institute, University of Sheffield, Sheffield, United Kingdoma; Department of Molecular Biology and Biotechnology, University of Sheffield, Sheffield, United Kingdomb; MRC Centre for Developmental and Biomedical Genetics, University of Sheffield, Sheffield, United Kingdomc; Department of Infection and Immunity, University of Sheffield, Sheffield, United Kingdomd; INRA, UMR1319 Micalis, Jouy-en-Josas, Francee; AgroParisTech, UMR Micalis, Jouy-en-Josas, Francef

## Abstract

Enterococcus faecalis is an opportunistic pathogen responsible for a wide range of life-threatening nosocomial infections, such as septicemia, peritonitis, and endocarditis. E. faecalis infections are associated with a high mortality and substantial health care costs and cause therapeutic problems due to the intrinsic resistance of this bacterium to antibiotics. Several factors contributing to E. faecalis virulence have been identified. Due to the variety of infections caused by this organism, numerous animal models have been used to mimic E. faecalis infections, but none of them is considered ideal for monitoring pathogenesis. Here, we studied for the first time E. faecalis pathogenesis in zebrafish larvae. Using model strains, chosen isogenic mutants, and fluorescent derivatives expressing green fluorescent protein (GFP), we analyzed both lethality and bacterial dissemination in infected larvae. Genetically engineered immunocompromised zebrafish allowed the identification of two critical steps for successful establishment of disease: (i) host phagocytosis evasion mediated by the Epa rhamnopolysaccharide and (ii) tissue damage mediated by the quorum-sensing Fsr regulon. Our results reveal that the zebrafish is a novel, powerful model for studying E. faecalis pathogenesis, enabling us to dissect the mechanism of enterococcal virulence.

## INTRODUCTION

Enterococcus faecalis is a Gram-positive commensal bacterium that colonizes the gastrointestinal tracts of humans and various animals. This bacterium is also responsible for a wide range of community- and hospital-acquired infections, including life-threatening bacteremia, infective endocarditis and peritonitis, and wound and urinary tract infections (1). The main risk factors for developing E. faecalis infections include impairment of the immune system, severe underlying diseases, urinary or vascular catheters, prior antibiotic therapy, and a prolonged stay in a hospital or intensive care unit (2). In some cases, diseases caused by E. faecalis are very difficult to treat, due to the large repertoire of intrinsic and acquired antibiotic resistance of this organism (3). Enterococci also represent a reservoir of genes conferring antibiotic resistance which can be disseminated to other pathogens, such as Staphylococcus aureus (4). Understanding the mechanisms of E. faecalis pathogenesis is therefore important to design preventive and alternative therapeutic approaches to treat enterococcal infections.

Several E. faecalis virulence factors have been identified using both mammalian [mainly mouse, rat, and rabbit (reviewed in reference 5)] and invertebrate [Caenorhabditis elegans (6), Galleria mellonella (7), and Drosophila melanogaster (8)] models of infection. These virulence factors are involved in attachment to host cells and extracellular matrix proteins, in cell and tissue damage and in immune system evasion (reviewed in references 3 and 9). Although invertebrates are amenable to large-scale genetic screens (10, 11), their physiology and immune systems differ significantly from mammalian systems. The zebrafish is a vertebrate organism that has been extensively used for developmental studies. The immune system of zebrafish shares with mammalian systems both innate and adaptive cellular immune system components (12), including macrophages and neutrophils already present at 1 day postfertilization (13). On a molecular level, high homology between zebrafish and human immune systems has also been observed, with Toll-like receptors (14, 15), nucleotide oligomerization domain receptors NOD1 and NOD2 (16), and the complement system (17, 18). In addition, since zebrafish larvae are optically transparent, most cell types, including macrophages (19) and neutrophils (20), can be microscopically imaged in real time upon inoculation of fluorescent bacteria into the host. As a genetically tractable organism that can be obtained in high numbers, the zebrafish combines several advantages of both invertebrate and rodent infection models. It has been used to study virulence in Salmonella enterica (21), Pseudomonas aeruginosa (22, 23), Burkholderia cenocepacia (24), S. aureus (25, 26), Listeria monocytogenes (27), and Streptococcus pneumoniae (28), among others. In addition, studies on a natural fish pathogen—Mycobacterium marinum—using embryonic and adult zebrafish have provided multiple new insights into granuloma formation and disease progression of tuberculosis (29). The zebrafish has thus become a model of choice for studying bacterial human diseases.

In this study, we explore the relevance of a zebrafish model of infection to study E. faecalis virulence. Using a combinatorial approach, targeting both host and pathogen components to dissect the complex host-pathogen interactions, we found that the zebrafish model of infection is particularly suitable for studying both E. faecalis-induced lethality and pathogenesis.

## MATERIALS AND METHODS

### Bacterial strains, plasmids, and growth conditions.

E. faecalis strains and plasmids are described in Table S1 in the supplemental material. Bacteria were grown in brain heart infusion (BHI) broth medium (Oxoid) at 37°C supplemented with antibiotics where appropriate at the following concentrations: tetracycline, 5 μg/ml; kanamycin, 2,000 μg/ml; and erythromycin, 30 μg/ml. E. faecalis strains were transformed with pMV158GFP (30) and pTEX5249 (31) by electroporation as previously described (31).

### Zebrafish maintenance and transgenesis.

London wild-type (LWT) inbred zebrafish embryos were provided by the aquarium staff of the MRC Centre for Developmental and Biomedical Genetics for zebrafish husbandry. The Tg(*mpeg1*:Gal4.VP-16)^sh256^ strain was generated as previously described (19). The Tg(*mpeg1*:Gal4.VP-16)^sh256^ strain was crossed to the Tg(UAS:*Kaede*)^s1999t^ strain to enable visualization of macrophages. Embryos were incubated in E3 medium at 28.5°C according to standard protocols (32).

### Morpholino knockdown of *pu.1.*

Morpholino-modified antisense oligomers against *pu.1* were injected as previously described (25).

### Microinjections of E. faecalis into zebrafish embryos.

Bacteria were grown in BHI broth until they reached an optical density at 600 nm of about 0.7 and harvested by centrifugation (5,500 × *g*, 10 min). Bacteria were microinjected into the circulation of dechorionated zebrafish embryos at 30 h postfertilization (hpf) as previously described (25). Briefly, anesthetized embryos were embedded in 3% (wt/vol) methylcellulose and injected individually using microcapillary pipettes filled with the bacterial suspension of known concentration. Larvae were observed frequently up to 90 h postinfection (hpi).

### Microscopic observations of larvae.

Live anesthetized larvae were immersed in 1% (wt/vol) low-melting-point agarose solution in E3 medium and mounted flat on a transparent slide. Images were acquired using the TE-2000U microscope (Nikon) with a Hamamatsu Orca-AG camera. Image acquisition and processing were performed with Volocity software (Improvision). A 4× Nikon Plan Fluor objective with a numerical aperture (NA) of 0.13 and a 60× Nikon Plan Apo oil objective with an NA of 1.4 were used.

### Determination of *in vivo* bacterial load.

At various times postinfection, six living zebrafish larvae were anesthetized and individually transferred with 100 μl of E3 medium into 0.5-ml Precellys tubes containing 1.4-mm ceramic beads (Peqlab) and homogenized using a Precellys 24-Dual homogenizer (Peqlab). The homogenates were serially diluted and plated on BHI agar to determine E. faecalis CFU numbers. Bacterial load was also determined for dead larvae at each time point.

### Phagocytosis assay.

Prior to infection, bacteria were labeled with pHrodo red succinimidyl ester (Invitrogen) by mixing 200 μl of bacterial suspension with 0.5 μl pHrodo red (2.5 mM) and incubating for 30 min in the dark. To remove the excess dye, bacteria were then washed with phosphate-buffered saline (PBS) followed by 50 mM Tris-HCl (pH 8.5) and subsequently resuspended in PBS. At 1.5 h postinfection (hpi), 18 larvae infected with each strain tested were mounted in 1% low-melting-point agarose, and microscopic images were captured using a 2× Nikon Plan UW objective (NA, 0.06) with a 543-nm excitation channel. The signal from pHrodo was then quantified for each embryo using ImageJ.

### Analysis of exoproteins.

E. faecalis strains were grown in BHI broth until exponential phase (optical density at 600 nm = 0.7) and harvested by centrifugation. Proteins in supernatants were precipitated by addition of trichloroacetic acid (TCA) at a 10% (wt/vol) final concentration. After incubation for 15 min on ice, proteins were recovered by centrifugation, and the protein pellet was washed with acetone. The final protein pellet was resuspended in Laemmli buffer prior to loading on a 12% (wt/vol) separation gel. The gel was stained with Coomassie blue R-250.

### Statistical analyses.

Survival experiments were evaluated using the Kaplan-Meier method. Comparisons between curves were made using the log rank test. The total pixel number in the phagocytosis assay was compared between two groups (OG1RF and *epaB*) using a two-tailed, unpaired Student's *t* test. Analysis was performed using Prism version 5.0 (GraphPad). Statistical significance was assumed at *P* values below 0.05.

## RESULTS

### Virulence of E. faecalis isolates in a zebrafish embryo infection model.

**Fig 1:**
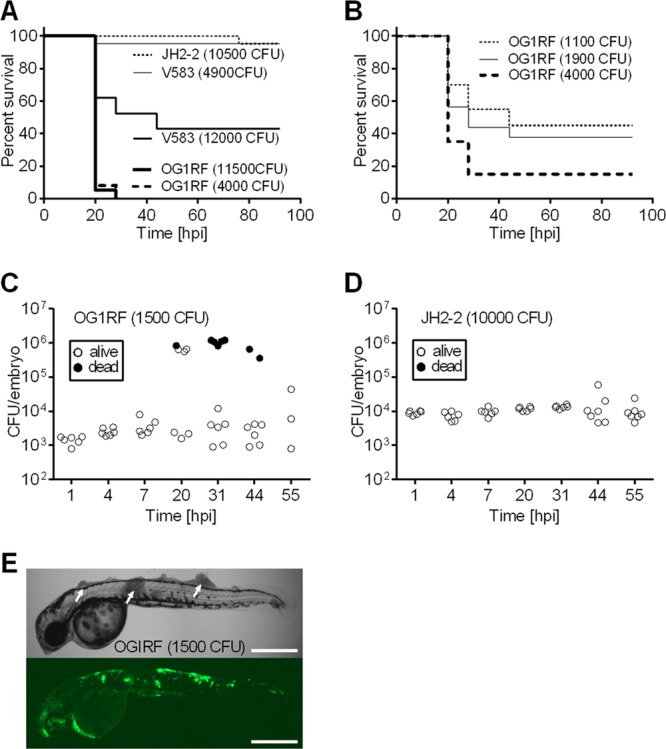
Intravenous infection of zebrafish embryos with E. faecalis OG1RF and V583 induces lethality, whereas infection with E. faecalis JH2-2 strain does not. (A) Survival of zebrafish larvae following injection with different doses of strains JH2-2, OG1RF, and V583 (*n* = 25). (B) Survival of zebrafish larvae following injection of various doses of E. faecalis OG1RF (*n* = 25). (C and D) Growth of E. faecalis OG1RF (C) and JH2-2 (D) within zebrafish larvae. (E) *In vivo* images of zebrafish larvae at 18 hpi following injection with GFP-labeled OG1RF. Bars, 500 μm. The numbers of CFU injected are indicated on each panel.

We analyzed the virulence of three E. faecalis reference strains in a zebrafish embryo infection model: OG1RF and V583, two clinical isolates extensively used to study E. faecalis pathogenesis, and JH2-2, a nonvirulent laboratory strain used for genetic studies. Each strain was injected into the bloodstream of LWT zebrafish embryos at 30 h postfertilization (hpf). Embryos were maintained at 28.5°C, and the survival of larvae was monitored for 90 h postinfection (hpi). Injection of high numbers of CFU (10,500 to 12,000) showed that the three strains displayed radically distinct virulence phenotypes (Fig. 1A and B): while JH2-2 killed only 10% of larvae at 90 hpi, OG1RF killed 100% of larvae by 24 hpi. V583 displayed an intermediate virulence, killing 50% of larvae at 42 hpi. Injection of different numbers of CFU revealed that killing by E. faecalis was dose dependent. OG1RF was more virulent than V583, as 4,000 CFU of this strain were able to kill approximately 80% of larvae at 24 hpi, whereas 4,900 CFU of V583 killed only 10% of larvae. Remarkably, OG1RF was still virulent at relatively low doses (1,100 CFU induced 50% mortality at 42 hpi).

The striking differences between OG1RF and JH2-2 prompted us to enumerate bacteria within larvae following infection. Embryos infected with sublethal doses of either OG1RF or JH2-2 strain were homogenized, and the numbers of bacteria within individual larvae were counted. In larvae infected with OG1RF, the number of bacteria increased slightly during the first 7 hpi. In some hosts, numbers did not increase, and larvae survived. In several larvae, CFU increased exponentially, reaching approximately 10^6^ CFU at 20 to 30 hpi, at which point death often occurred (Fig. 1C). The variable number of CFU from larva to larva may reflect the variability between individual hosts. Alternatively, we can speculate that high CFU numbers reflect a more advanced infection, leading to death when CFU numbers reach 10^5^ to 10^6^ per larva. In contrast, strain JH2-2 was unable to proliferate *in vivo*, and the bacterial numbers remained constant until at least 55 hpi (Fig. 1D).

Taking advantage of the embryonic optical transparency of developing zebrafish, we monitored bacterial dissemination during infection. For comparison purposes, both OG1RF and JH2-2 were transformed with a replicative plasmid constitutively expressing *gfp*. Zebrafish larvae were infected with one of the two E. faecalis-transformed strains and analyzed by fluorescence microscopy. Larvae infected with OG1RF developed lesions resembling necrotic tissues at sites of bacterial growth, referred to here as “tissue damage” (Fig. 1E, arrows). In contrast, the JH2-2-infected larvae remained apparently normal, with bacteria being restricted to small foci within the embryonic vasculature (data not shown). The OG1RF strain was able to disseminate systemically and proliferate within larvae and was often detected in the heart and central nervous system prior to embryo death (see Fig. S1A in the supplemental material). Zebrafish larvae that survived the infection were not able to completely clear bacteria from their bodies, and small foci of infection were seen until at least 90 hpi (see Fig. S1B).

### Contribution of phagocytes to E. faecalis infection.

**Fig 2:**
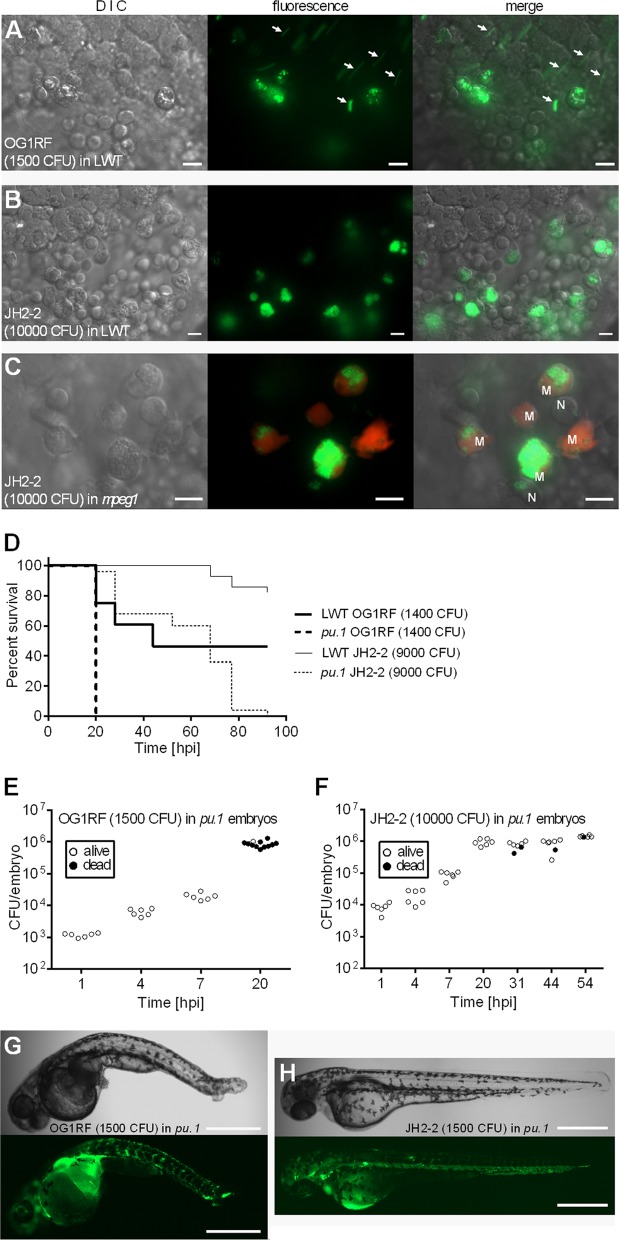
Differential uptake of OG1RF and JH2-2 strains by phagocytes plays an important but not exclusive role in killing. (A and B) *In vivo* images of the yolk circulation valley of embryos at 2 hpi following injection with OG1RF (A) and JH2-2 (B) strains expressing *gfp*. Arrows indicate examples of unphagocytosed bacteria. These appear as “smears” due to their movement in the bloodstream and the relatively long exposure time. (C) *In vivo* imaging of the yolk circulation valley of Tg(*mpeg1*:Gal4 × UAS:*Kaede*) transgenic embryos at 2 hpi injected with GFP-expressing JH2-2. Photoconverted Kaede-positive macrophages (M) and nonfluorescent neutrophils (N) containing fluorescent E. faecalis are indicated. (D) Survival of wild-type or phagocyte-depleted zebrafish larvae following injection with E. faecalis OG1RF or JH2-2 (*n* = 25). (E and F) Growth of E. faecalis within phagocyte-depleted zebrafish larvae upon injection with OG1RF (E) or JH2-2 (F) strain into the bloodstream. (G and H) *In vivo* images of phagocyte-depleted zebrafish larvae injected with GFP-expressing OG1RF (G) or GFP-expressing JH2-2 at 18 hpi (H). Bars, 10 μm (A, B, and C) and 500 μm (G and H). The numbers of CFU injected are indicated on each panel.

At 30 hpf, embryo immune defenses rely on the innate response, primarily macrophages and neutrophils. To gain insight into the differences in virulence of OG1RF and JH2-2, we compared bacterial uptake by phagocytes at 2 hpi. In zebrafish larvae infected with 1,500 CFU of fluorescent E. faecalis OG1RF, a large proportion of fluorescent bacteria resisted phagocytosis and remained free in the bloodstream (Fig. 2A, arrows). In contrast, JH2-2 bacteria injected at a much higher dose (10,000 CFU) were all promptly phagocytosed (Fig. 2B).

To determine specific macrophage roles in engulfing injected enterococci, we used embryos expressing the macrophage-specific transgenes *mpeg1*:Gal4 and UAS:*Kaede* (19). At 29 hpf, embryos were subjected to 436 nm irradiation for 20 min to enable Kaede photoconversion from green to red. The embryos were then infected with *gfp*-expressing E. faecalis JH2-2. Similarly to previous studies with other bacteria (25, 33), the majority of fluorescent enterococci were detected within macrophages (Fig. 2C).

To further explore the role of zebrafish phagocytes in resistance to E. faecalis infection, phagocytes were depleted using antisense *pu.1* morpholinos. *pu.1* morphants were significantly more susceptible to both OG1RF and JH2-2 than their immunocompetent counterparts (Fig. 2D). However, while OG1RF rapidly killed all phagocyte-depleted larvae, JH2-2 required 3 to 4 days to kill immunocompromised larvae. Despite the distinct death kinetics associated with strains OG1RF and JH2-2, both were able to proliferate rapidly in *pu.1* morphants, reaching approximately 10^6^ CFU per embryo at 20 hpi (Fig. 2E and F). Imaging of *pu.1* morphants when bacterial proliferation was maximal showed serious deformation and tissue damage in OG1RF-infected larvae (Fig. 2G), whereas larvae infected with JH2-2 showed no signs of disease despite heavy dissemination (Fig. 2H). This result suggested that in addition to being easily phagocytosed, the JH2-2 strain lacks virulence determinants that contribute to killing of the host. Interestingly, this work indicates that zebrafish larvae represent a tractable model for studying E. faecalis pathogenesis under conditions that mimic neutropenia, a major risk factor for E. faecalis infections.

### Contribution of *epa*, *fsr*, *gelE*, and *sprE* to E. faecalis pathogenesis.

To gain further insights into the zebrafish model of E. faecalis infection, we analyzed OG1RF mutants previously shown to be attenuated in other animal models. Based on our results, which suggested a role of phagocytosis and revealed substantial tissue damage following infection, we focused on two types of virulence factors: (i) the enterococcal polysaccharide antigen (encoded by the *epa* locus), a rhamnopolysaccharide that has previously been shown to play a role in resistance to neutrophil uptake *in vitro* (34), and (ii) two major exoproteases playing a role in virulence, a gelatinase (GelE) and a serine protease (SprE) that are regulated by the Fsr quorum-sensing system (7, 31, 35). Four OG1RF mutants (*epaB*, *gelE*, *sprE*, and *fsr* mutants) were tested in our infection model.

**Fig 3:**
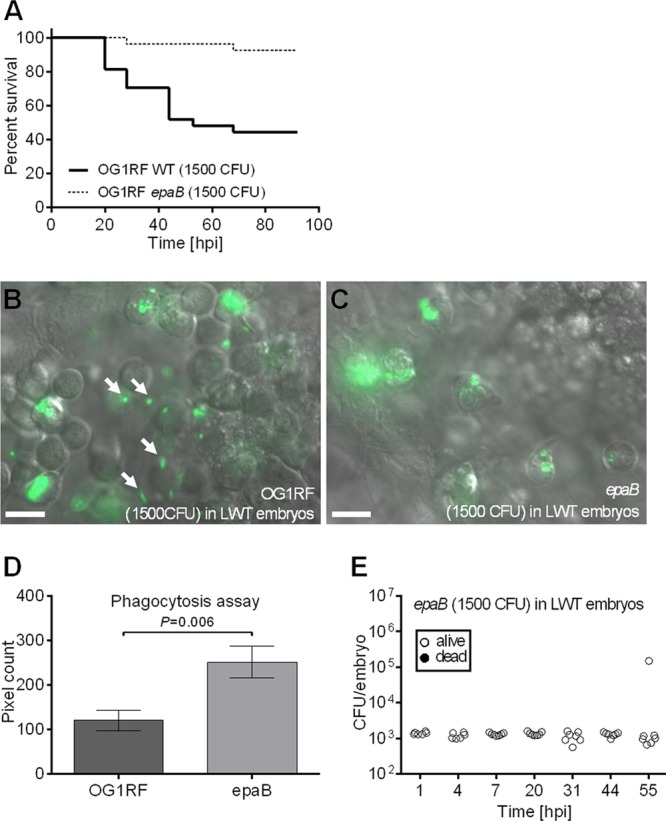
Enterococcal polysaccharide antigen (*epa*) mutants of OG1RF are unable to evade phagocytosis and fail to establish lethal infection. (A) Survival of zebrafish larvae following injection with E. faecalis OG1RF and *epa* derivatives (*n* = 25). In log rank tests for *epaB* versus wild type, *P* is 0.0001. (B and C) *In vivo* images of the yolk circulation valley of larvae at 2 hpi following injection with *gfp*-expressing OG1RF (B) or the *epaB* mutant (C). Arrows indicate examples of unphagocytosed bacteria. (D) Efficacy of phagocytosis by zebrafish embryos (*n* = 20) at 1.5 h following infection with pHrodo-labeled wild-type E. faecalis OG1RF or the *epaB* mutant. (E) Growth of the *E. faecalis epaB* mutant in zebrafish larvae. The numbers of CFU injected are indicated on each panel. Bars, 10 μm.

The *epaB* mutant was significantly attenuated compared to the parental strain (Fig. 3A) (*P* value of 0.0001). To investigate the role of Epa in phagocytosis *in vivo*, we carried out microscopic examination of larvae infected with *epaB* mutant expressing green fluorescent protein (GFP). Unlike the parental strain (Fig. 3B), the *epaB* mutant was not able to evade phagocytosis *in vivo* and was readily taken up by zebrafish phagocytes compared to the wild-type strain (Fig. 3C). The difference in phagocytic uptake between OG1RF and its *epaB* derivative was quantified following labeling of bacterial cells with pHrodo-S-ester, a pH-sensitive dye that enables us to visualize bacteria only in low-pH compartments of phagosomes (36). This assay confirmed that at 1.5 hpi, the *epaB* mutant is phagocytosed more efficiently than the parental strain (Fig. 3D). Bacterial counts following infection with the *epaB* mutant remained constant until 55 hpi (Fig. 3E), clearly showing that although growth was abolished in zebrafish larvae, *epaB*-deficient bacteria were still able to survive within phagosomes. Together, our results therefore indicate that the Epa rhamnopolysaccharide contributes to evasion of phagocytosis but does not contribute to survival in zebrafish phagosomes.

**Fig 4:**
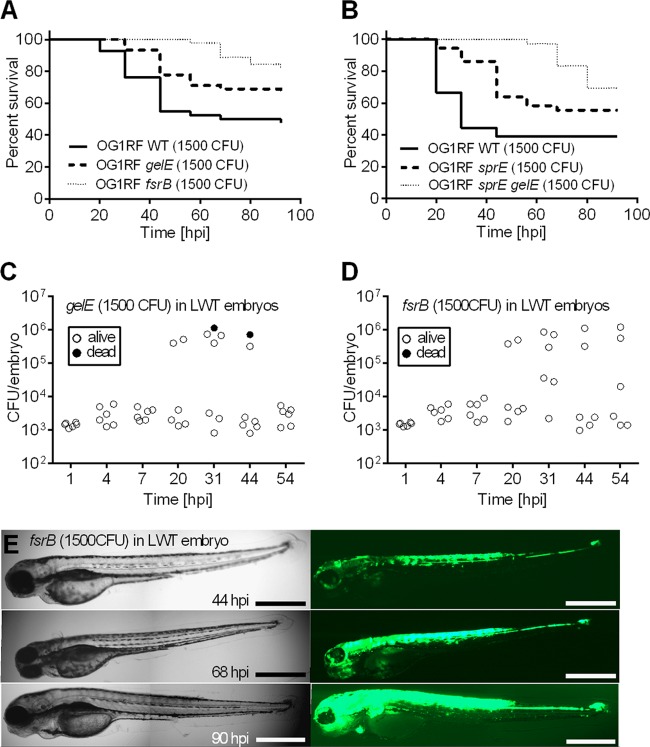
Protease-deficient mutants of OG1RF proliferate *in vivo* but are less virulent than the wild type. (A and B) Survival of zebrafish larvae (*n* ≥ 36) following injection with the indicated E. faecalis mutant strains. In log rank tests, for the *gelE* mutant versus the wild type (WT), *P* = 0.04; for the *fsrB* mutant versus the WT, *P* = 0.0001; for the *sprE* mutant versus the WT, *P* = 0.03; for the *sprE gelE* double mutant versus the WT, *P* = 0.0006. (C and D) Growth of the *E. faecalis gelE* (C) or *fsrB* (D) mutant in zebrafish larvae (E). Time-lapse microscopy of a representative zebrafish larva infected with a GFP-expressing *E. faecalis fsrB* mutant. The numbers of CFU injected are indicated on each panel. Bars, 500 μm.

In our model, *gelE*, *sprE*, and *fsr* mutants were significantly attenuated compared to the parental strain (Fig. 4A and B) (*P* values of 0.04, 0.03, and 0.0001 for *gelE*, *sprE*, and *fsr* mutants, respectively). Both *gelE* and *fsrB* mutants were selected to be further analyzed *in vivo*, as two examples of intermediate or severe reduction of virulence, respectively. Unexpectedly, both attenuated strains were able to proliferate similarly to the parental strain (Fig. 4C and D), although this growth did not result in larva death. In agreement with this observation, time-lapse imaging of larvae infected with *fsrB* mutants expressing *gfp* did not reveal any morphological change, even when the majority of the developing zebrafish were overwhelmed with bacteria (Fig. 4E). This observation indicates that the Fsr system is not required for *in vivo* proliferation but contributes to late stages of infection.

**Fig 5:**
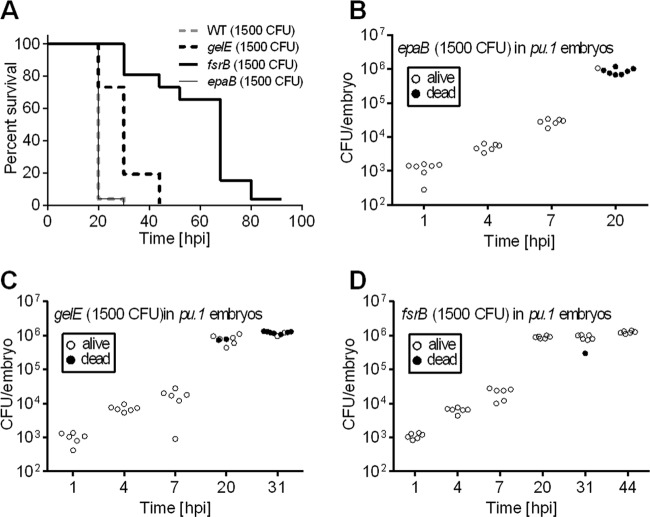
Protease-deficient mutants of OG1RF remain attenuated in phagocyte-depleted larvae. (A) Survival of *pu.1* knockdown zebrafish larvae (*n* = 25) following infection with *E. faecalis fsrB*, *gelE*, and *epaB* mutants. In log rank tests, for *gelE* versus WT, *P* < 0.0001; for *fsrB* versus WT, *P* < 0.0001; for *epaB* versus WT, *P* = 0.97. (B to D) Growth of *E. faecalis epaB* (B), *gelE* (C), and *fsrB* (D) mutants in *pu.1* knockdown larvae.

To determine whether the attenuation of the mutants analyzed was caused by their inability to overcome the host cellular immune response, we followed the infection of phagocyte-depleted larvae (Fig. 5). As expected, the virulence of *epaB* mutant was fully restored in *pu.1* morphants. In contrast, death of larvae infected with the *gelE* mutants was significantly delayed compared to that of the parental strain. For *pu.1* morphants infected with the *fsrB* mutant, the time to death was severely delayed (Fig. 5A). Interestingly, bacterial growth in larvae was very similar for all mutants tested, reaching approximately 10^6^ CFU at 20 hpi (Fig. 5B to D). However, different death rates indicated that *in vivo* proliferation is not sufficient to cause death. Altogether, these results indicate that zebrafish represent a particularly suitable model for following two steps crucial to the progression of E. faecalis pathogenesis: phagocytosis evasion and host killing, in part mediated by exoprotease genes and other *fsrB*-dependent genes.

### Heterologous complementation of the *fsr* locus in JH2-2.

**Fig 6:**
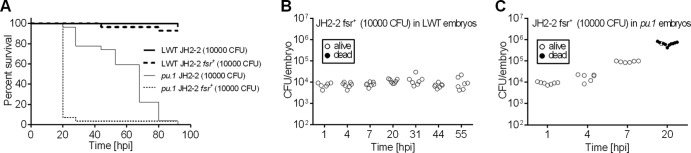
Heterologous expression of OG1RF *fsr* locus in JH2-2 restores virulence in phagocyte-depleted but not wild-type larvae. (A) Survival of wild-type or *pu.1* knockdown zebrafish larvae (*n* = 25) following infection with E. faecalis JH2-2 with or without *fsrB* complementation. In log rank tests, for JH2-2 versus JH2-2 *fsrB*^+^ in wild-type larvae, *P* = 0.56; for JH2-2 versus JH2-2 *fsrB*^+^ in *pu.1* knockdown larvae, *P* < 0.0001. (B and C) Growth of E. faecalis JH2-2 *fsr*^+^ in wild-type (B) or *pu.1* knockdown (C) larvae. The numbers of CFU injected are indicated on each panel.

Survival of *pu.1* knockdown larvae infected with the OG1RF *fsrB* mutant (Fig. 5A) was very similar to that of immunocompromised larvae infected with JH2-2 (Fig. 2D). We hypothesized that the inactive *fsr* system in JH2-2 could at least in part account for the delay in mortality in immunocompromised larvae. To test this hypothesis, we complemented the JH2-2 strain with the OG1RF *fsr* locus using plasmid pTEX5249 (31). As anticipated, heterologous expression of the OG1RF *fsr* locus restored the production of exoproteases in JH2-2 (37) (see Fig. S2 in the supplemental material). JH2-2 *fsr*^+^ bacteria were avirulent when injected into immunocompetent larvae but rapidly killed phagocyte-depleted larvae (Fig. 6A). Analysis of bacterial load following infection revealed no growth of JH2-2 *fsr*^+^ bacteria in wild-type larvae (Fig. 6B), indicating that restoration of neither exoprotease production nor any other Fsr-dependent factor allowed JH2-2 to overcome phagocytosis. In *pu.1* knockdown larvae, JH2-2 *fsr*^+^ bacteria proliferated rapidly and caused death of infected larvae (Fig. 6C), highlighting the important role of Fsr-dependent factors, including exoproteases, in later stages of infection, after the initial immune hurdle (i.e., phagocytes) has been overcome. These results show that the genetically manipulable strain JH2-2 can be used as a platform strain to investigate functions of virulence factors.

## DISCUSSION

Several vertebrate models for studying specific E. faecalis infections have been described: endophthalmitis and endocarditis in rabbits and urinary tract infection, bacteremia, and peritonitis in mice (5). Although these models have been successfully used to characterize a large variety of E. faecalis virulence factors, they are not adapted for large-scale studies and remain costly. Over the past decade, zebrafish have been successfully used as a surrogate model host for human bacterial pathogens (38). In this study, we showed that zebrafish can be used as a host to study E. faecalis pathogenesis. Our results revealed that this infection model is particularly suitable for identifying E. faecalis virulence factors, dissecting the mechanisms of infection, and following the dissemination of bacteria *in vivo*.

### Zebrafish as a model for studying E. faecalis virulence.

This work showed that zebrafish larvae are susceptible to virulent E. faecalis strains (OG1RF and V583) in a dose-dependent manner but resistant to strain JH2-2, a nonvirulent laboratory strain. In agreement with previous studies comparing OG1RF and V583 in mouse and C. elegans models (6, 39), we also show that OG1RF was more virulent than V583 in the zebrafish lethality model (Fig. 1). This work also confirms the poor pathogenic potential of JH2-2. When high numbers of CFU of these bacteria were injected, they failed to induce lethality despite the fact that they were able to multiply and persist *in vivo*.

To demonstrate the relevance of the zebrafish model of infection, we focused on the analysis of mutations in the protease genes *gelE* and *sprE* and the *fsr* quorum-sensing system, which positively controls their expression (31). Mutation in the *fsr* locus has been associated with a significant decrease in virulence in all animal models tested (7, 31, 35, 40). Similarly to previous studies on various animal models, we found that *fsrB* deletion resulted in severe loss of enterococcal virulence in zebrafish larvae. However, we showed that the *fsrB* mutant was able to proliferate as well as its parent strain, OG1RF (Fig. 4), indicating that *fsrB* controls factors important for the late stages of infection, leading to killing. While GelE has been unambiguously demonstrated to be important for enterococcal virulence in multiple models (31, 35, 41–43), the importance of SprE as an E. faecalis virulence factor is controversial. The *sprE* mutant was shown to be attenuated in mouse peritonitis in the nematode and the fly models (8, 31, 35). However, SprE was dispensable for rabbit infective endocarditis and had no lethal activity in Galleria mellonella (42, 43). The role of these exoproteases in promoting tissue damage is strongly supported based on histopathological examination of infected eyes using the endophthalmitis model (44) and their proteolytic activity over a wide substrate range (45). Here, we showed that both the gelatinase GelE and the serine protease SprE contribute to E. faecalis virulence in zebrafish larvae and appear to have additive effects in pathogenesis. Altogether, given that E. faecalis is able to proliferate and disseminate within zebrafish larvae, this infection model appears to be suitable for identifying a range of virulence factors associated with E. faecalis pathogenesis. One potential limitation of the zebrafish infection model is the temperature at which the larvae are kept (28.5°C). Thus, alternative infection models need to be considered to analyze virulence factors specifically expressed at 37°C, the temperature of the mammalian host.

### Zebrafish as a model system to study innate immune response against E. faecalis.

In this study, we showed that zebrafish larvae can be used as a model to evaluate both uptake and survival of bacteria in phagocytes. Upon injection into the bloodstream, E. faecalis phagocytosis is mediated by macrophages. This result is in agreement with previous studies indicating that in zebrafish, bacteria in blood circulation are essentially phagocytosed by macrophages, neutrophils being able to engulf only surface-associated microbes (46). Although macrophages completely phagocytosed a large inoculum of JH2-2, a significant proportion of OG1RF cells escaped phagocytosis and remained in the bloodstream. E. faecalis-specific rhamnopolysaccharide encoded by the *epa* locus was previously shown to mediate resistance to uptake and killing by human neutrophils (11, 34). Using the Epa-deficient *epaB* mutant (47), we showed that the Epa rhamnopolysaccharide protects OG1RF from uptake by macrophages in zebrafish. In the phagocyte-depleted larvae, the *epaB* mutant was as virulent as the parental OG1RF strain, indicating that during infection, the main role of the *epa* cluster is to prevent phagocytosis. However, our data indicate that the rhamnopolysaccharide encoded by the JH2-2 *epa* locus is not able to confer resistance to phagocytosis *in vivo*. Diversity of the *epa* locus within E. faecalis species was recently reported (48). The zebrafish model is particularly appropriate for evaluating whether Epa diversity impacts E. faecalis resistance to phagocytosis *in vivo. E. faecalis* persistence within zebrafish larvae is in agreement with the extreme resistance of this bacterium to various stresses (reviewed in reference 49). It will be interesting to test whether factors that have been shown to enhance survival in murine macrophages (50) or human neutrophils (51) play a role in resistance to zebrafish macrophages. Neutropenia is a major risk factor for enterococcal infections (2). The higher susceptibility of immunocompromised zebrafish larvae to E. faecalis infection suggests that this model is suitable for investigating E. faecalis pathogenesis under conditions mimicking disease onset.

### Concluding remarks.

Our study revealed that zebrafish larvae represent an attractive alternative model system for studying host-pathogen interaction during E. faecalis infections, allowing the identification of both host immune components and pathogen virulence factors. This novel vertebrate model enables spatial and temporal dissection of the complex mechanisms of E. faecalis infection *in vivo*, from initial interaction with host innate immune system to development of bacterial quorum sensing *in vivo* and degradation of host tissues, resulting in death. We showed that the degree of virulence can be readily measured by a simple lethality assay. More importantly, thanks to zebrafish optical transparency and genetic tractability, we also reveal that successful infection by E. faecalis relies on two major steps: phagocyte evasion and tissue damage. Based on these observations, we are confident that the model described here will be useful for large-scale screening of mutant libraries to identify new bacterial virulence determinants and host factors important in immunity against enterococci.

## Supplementary Material

Supplemental material
