# Supplementary material for: Zebrafish as a Novel Vertebrate Model To Dissect Enterococcal Pathogenesis
Source: Infect Immun. 2013 Nov;81(11):4271–9. doi: 10.1128/IAI.00976-13 (PMC3811811; doi:10.1128/IAI.00976-13)
Supplement: Supplemental material [file supp_81_11_4271__index.html]

Zebrafish as a Novel Vertebrate Model To Dissect Enterococcal Pathogenesis — Supplemental material 

# Zebrafish as a Novel Vertebrate Model To Dissect Enterococcal Pathogenesis

## Supplemental material

**Files in this Data Supplement:**

- Supplemental file 1 -

  Fig. S1. *In vivo* time-lapse microscopy of zebrafish larvae infected with 1,500 CFU of GFP-expressing *E. faecalis* OG1RF into the bloodstream. Fig. S2. Exoprotein profiles from *E. faecalis* strains OG1RF and JH2-2. Table S1. Strains and plasmids used in this study.

  PDF, 6.3M
