# Supplementary material for: Zebrafish as a Novel Vertebrate Model To Dissect Enterococcal Pathogenesis
Source: Infect Immun. 2013 Nov;81(11):4271–9. doi: 10.1128/IAI.00976-13 (PMC3811811; doi:10.1128/IAI.00976-13)
Supplement: Supplemental material [file IAI.00976-13_zii999090389so1.pdf]

Figure S1.

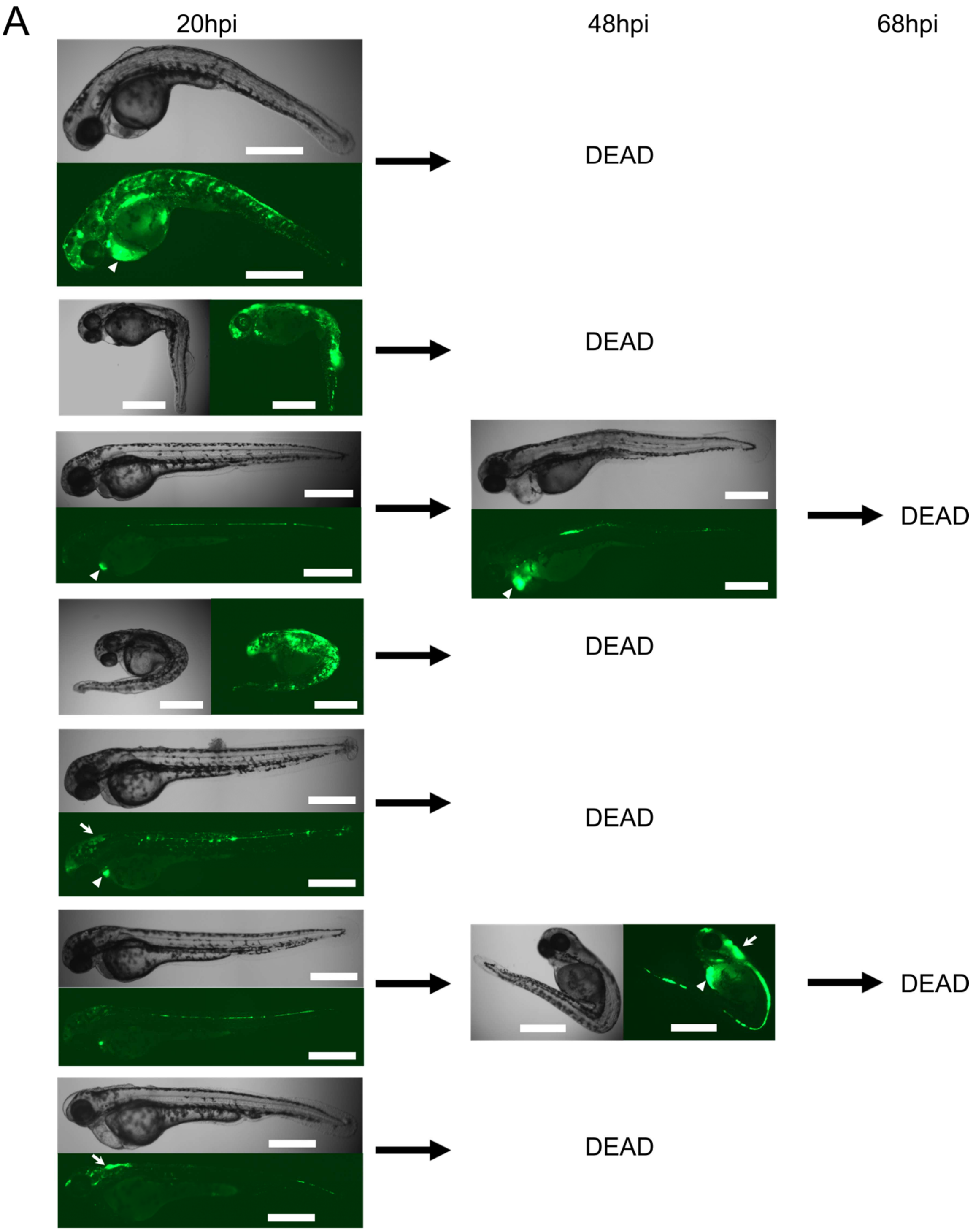

Figure S1.

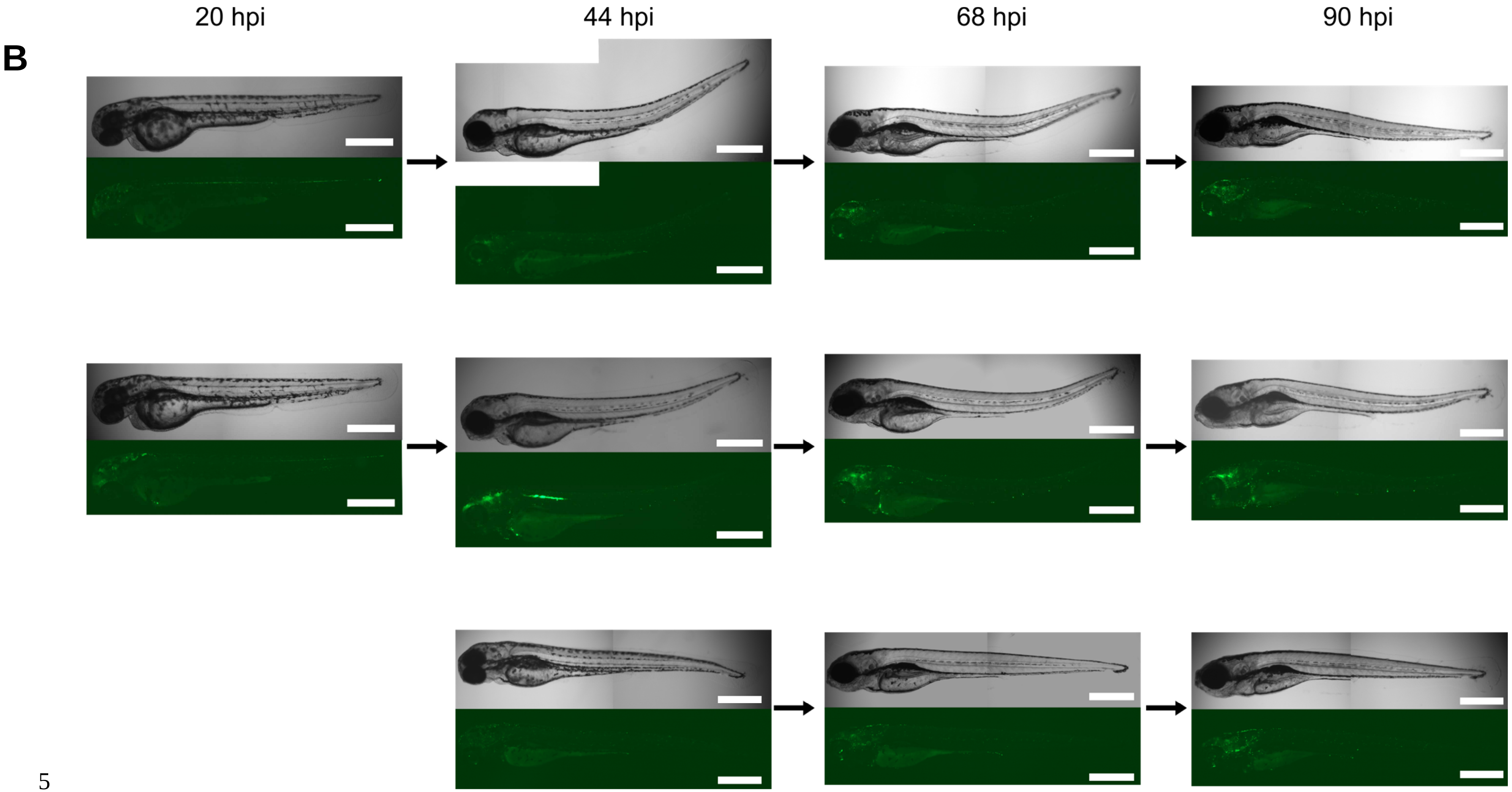

Figure S2.

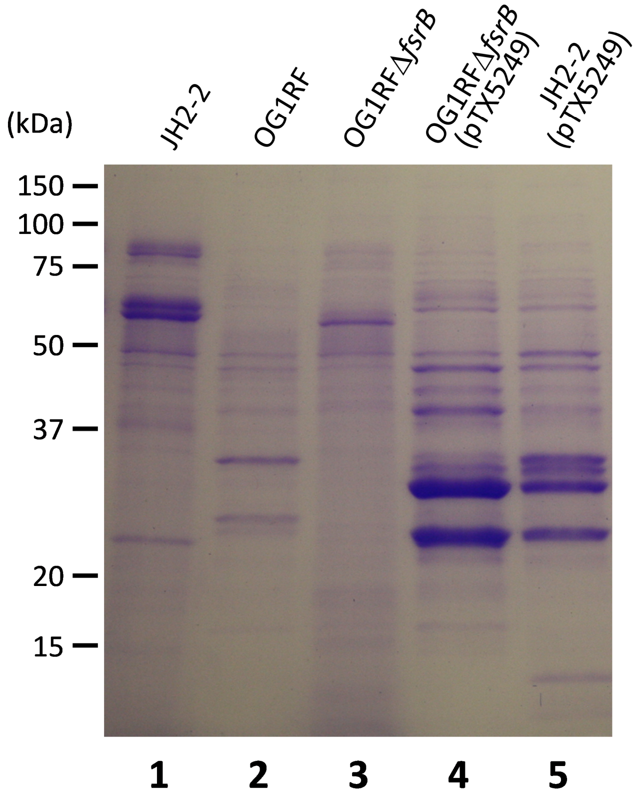

Figure S1. ***In vivo* time-lapse microscopy of zebrafish larvae infected with 1500 CFUs of GFP-expressing *E. faecalis* OG1RF into the bloodstream.** Panel A shows examples of infected zebrafish larvae succumbing to *E. faecalis* infection, whereas larvae resisting the disease are shown in panel B. Arrowheads indicate heart, arrows indicate central nervous system. Scale bars represent 500 µm.

Figure S2**. Exoprotein profiles from *E. faecalis* strains OG1RF and JH2-2.** Exponential phase culture supernatants were precipitated with TCA 10% (w/v) and analyzed by SDS-PAGE. Lane 1, JH2-2; lane 2, OG1RF; lane 3, OG1RF *fsrB*; lane 4, OG1RF *fsrB* (pTEX5249); lane 5, JH2-2 (pTEX5249).

Table S1. Strains and plasmids used in this study

Strain/plasmid	Genotype or description	Reference
Strain		
JH2-2	Plasmid-free laboratory strain	(1)
JH2-2-GFP	JH2-2 carrying pMV158GFP	This study
JH2-2 *fsr*$^{+}$	JH2-2 carrying pTEX5249	This study
V583	Clinical isolate	(2)
OG1RF	Clinical isolate	(3)
OG1RF-GFP	OG1RF carrying pMV158GFP	This study
OG1RF *gelE*	TX5264; *gelE* deletion mutant	(4)
OG1RF *sprE*	TX5243; *sprE* insertion mutant	(5)
OG1RF Δ(*gelE-sprE)*	JRC105; OG1RF derivative harbouring a deletion of the *gelE-sprE* operon	(6)
OG1RF *fsrB*	TX5266; *fsrB* deletion mutant	(7)
OG1RF *fsrB* GFP	OG1RF *fsrB* mutant carrying pMV158GFP	This study
OG1RF *epaB*	TX5179 mutant harbouring an insertion in *epaB* (formerly *orfde4*)	(8)
OG1RF *epaB* GFP	OG1RF *epaB* mutant carrying pMV158GFP	This study
Plasmid		
pMV158GFP	Replicative plasmid for constitutive expression of GFP	(9)
pTEX5249	Replicative plasmid carrying the OG1RF *fsrABC* locus	(5)

References

1. **Jacob AE, Hobbs SJ**. 1974. Conjugal transfer of plasmid-borne multiple antibiotic resistance in *Streptococcus faecalis* var. *zymogenes*. J. Bacteriol. **117**:360–372.
2. **Paulsen IT, Banerjei L, Myers GSA, Nelson KE, Seshadri R, Read TD, Fouts DE, Eisen JA, Gill SR, Heidelberg JF, Tettelin H, Dodson RJ, Umayam L, Brinkac L, Beanan M, Daugherty S, DeBoy RT, Durkin S, Kolonay J, Madupu R, Nelson W, Vamathevan J, Tran B, Upton J, Hansen T, Shetty J, Khouri H, Utterback T, Radune D, Ketchum KA, Dougherty BA, Fraser CM**. 2003. Role of mobile DNA in the evolution of vancomycin-resistant *Enterococcus faecalis*. Science **299**:2071–2074.
3. **Bourgogne A, Garsin DA, Qin X, Singh KV, Sillanpaa J, Yerrapragada S, Ding Y, Dugan-Rocha S, Buhay C, Shen H, Chen G, Williams G, Muzny D, Maadani A, Fox KA, Gioia J, Chen L, Shang Y, Arias CA, Nallapareddy SR, Zhao M, Prakash VP, Chowdhury S, Jiang H, Gibbs RA, Murray BE, Highlander SK, Weinstock GM**. 2008. Large scale variation in *Enterococcus faecalis* illustrated by the genome analysis of strain OG1RF. Genome Biol. **9**:R110.
4. **Sifri CD, Mylonakis E, Singh KV, Qin X, Garsin DA, Murray BE, Ausubel FM, Calderwood SB**. 2002. Virulence effect of *Enterococcus faecalis* protease genes and the quorum-sensing locus *fsr* in *Caenorhabditis elegans* and mice. Infect. Immun. **70**:5647–5650.
5. **Qin X, Singh KV, Weinstock GM, Murray BE**. 2000. Effects of *Enterococcus faecalis fsr* Genes on Production of Gelatinase and a Serine Protease and Virulence. Infect. Immun. **68**:2579–2586.
6. **Kristich CJ, Chandler JR, Dunny GM**. 2007. Development of a host-genotype-independent counterselectable marker and a high-frequency conjugative delivery system and their use in genetic analysis of *Enterococcus faecalis*. Plasmid **57**:131–144.
7. **Qin X, Singh KV, Weinstock GM, Murray BE**. 2001. Characterization of *fsr*, a regulator controlling expression of gelatinase and serine protease in *Enterococcus faecalis* OG1RF. J. Bacteriol. **183**:3372–3382.
8. **Xu Y, Singh KV, Qin X, Murray BE, Weinstock GM**. 2000. Analysis of a gene cluster of *Enterococcus faecalis* involved in polysaccharide biosynthesis. Infect. Immun. **68**:815–823.
9. **Nieto C, Espinosa M**. 2003. Construction of the mobilizable plasmid pMV158GFP, a derivative of pMV158 that carries the gene encoding the green fluorescent protein. Plasmid **49**:281–285.
